# Remedies and Their Uses

**Published:** 1906-07-14

**Authors:** 


### Remedies and their Uses.

### Strophanthin.

Kakowski in the paper quoted above noted a marked difference between strophanthus preparations and those of digitalis. He found that whereas a fuller effect was obtained with infusion of digitalis than with any of its constituent glucosides, with strophanthus the active principle strophanthin was superior in its action to the Pharmacopœial tincture. Strophanthus itself has been long known. The seeds of a species of strophanthus were first introduced into Europe in 1865, having been obtained from East and West Africa, where they were used by the natives for the preparation of arrow poison. It was not, however, till 1890 that Sir Thomas Fraser drew attention to the pharmacological properties of strophanthus seeds as a cardiac tonic. The very unequal results reported clinically of strophanthus have been shown to depend partly on the species of plant from the seeds of which the alcoholic tincture is prepared. It was found impossible to distinguish between varieties of strophanthus seeds as imported. Strophanthus kombe from South East Africa is now the only official variety in Germany, but not only has it proved impossible to distinguish the seeds from other kinds, but the difficulties of obtaining the active principle strophanthin in a pure crystalline state are almost insuperable. Gilg, however, has recently shown that the seeds of strophanthus gratus can be positively and easily distinguished from those of other varieties. He has described their botanical characters, and shown that, whereas the split seeds of other varieties of strophanthus strike a green colour with sulphuric acid, those of S. gratus give a reddish tint changing to violet. Thoms has succeeded in preparing strophanthin by simple chemical means from these seeds in a pure crystalline state. This preparation gives the characteristic red colour with sulphuric acid, has a definite melting point, and is said to be identical with Ouabaine. It is a stable substance, easily soluble in hot water and amyl alcohol. Schedel[7] states that this preparation known as G. strophanthin has a rapid and certain physiological action, that untoward symptoms are less frequent with its use, and it is less rapidly cumulative than digitalis. It is used in 1 per cent. watery solution, the dose being one or two drops. Its solubility renders it suitable for hypodermic use, while its chemical purity ensures uniformity of action.

Both G. strophanthin and digalin have the advantage of being pure chemical products; the former is superior in its action to the usual tincture, but the latter may by reason of its purity miss some of the valuable therapeutic properties of infusions of the fresh leaves. It is, however, a far more reliable drug than the pharmacopœial infusion and tincture, which may, from accidental causes, be practically without physiological effect, and thus will commend itself in practice, even if farther experience should show that all the advantages claimed for it over digitalis leaves are not invariably exhibited. At present its main disadvantages appear to be its somewhat high price, and its unsuitability for hypodermic use.

[1] Zeitsch. f. Krankenpflege, xxvii. 7, 1905.

